# Effects of chronic treatment with new strains of *Lactobacillus plantarum* on cognitive, anxiety- and depressive-like behaviors in male mice

**DOI:** 10.1371/journal.pone.0234037

**Published:** 2020-06-19

**Authors:** Thaísa Barros-Santos, Kallyane Santos Oliveira Silva, Matheus Libarino-Santos, Elisangela Gouveia Cata-Preta, Henrique Sousa Reis, Eduardo Koji Tamura, Alexandre Justo de Oliveira-Lima, Laís Fernanda Berro, Ana Paula Trovatti Uetanabaro, Eduardo Ary Villela Marinho

**Affiliations:** 1 Department of Biological Sciences, Universidade Estadual de Santa Cruz, Ilhéus, Brazil; 2 Department of Health Sciences, Universidade Estadual de Santa Cruz, Ilhéus, Brazil; 3 Department of Psychiatry and Human Behavior, University of Mississippi Medical Center, Jackson, Mississippi, United States of America; Medizinische Universitat Graz, AUSTRIA

## Abstract

Psychobiotics correspond to a class of probiotics, mainly of the genus *Lactobacillus* and *Bifidobacterium*, capable of producing neuroactive substances, such as γ-aminobutyric acid (GABA) and serotonin, which exert effects on the brain-gut axis. Evidence suggests that psychobiotics can have a beneficial effect on mood, anxiety and cognition. The present study evaluated the effects of chronic administration of two new strains of *Lactobacillus plantarum*, *L*. *plantarum* 286 (Lp 286) and *L*. *plantarum* 81 (Lp 81) isolated from the fermentation of cocoa (*Theobroma cacao* L.) and cupuaçu (*Theobroma grandiflorum*), respectively, on cognitive, anxiety- and depressive-like behaviors in male Swiss mice. Different groups of animals were administered (oral gavage) solutions of vehicle (0.85% saline plus 15% skim milk), Lp 286 (10^9^/0.1 ml CFU) or Lp 81 (10^9^/0.1 ml CFU) for 30 days, and animals were tested for general locomotor activity, depressive-like behavior in the forced swim test, and learning/memory and anxiety-like behavior in the plus-maze discriminative avoidance task. Treatment with the strains Lp 286 and Lp 81 did not interfere with locomotor activity or learning and memory. The Lp 286 strain exerted anti-depressant- and anxiolytic-like effects under our experimental conditions. Our findings add to the current body of evidence suggesting that probiotics from the genus *Lactobacillus* may exert psychobiotic potential and introduce a new strain, Lp 286, as a potential candidate in the prevention or as therapeutic adjuvant in the treatment of mental disorders.

## Introduction

The use of microorganisms capable of manipulating the intestinal microbiota has gained visibility for the promotion of several health benefits. The World Health Organization [1] regulates and defines probiotics as living microorganisms which, when administered in appropriate amounts, confer a beneficial effect to the host’s health. Species of the groups *Lactobacilli* and *Bifidobacteria* are the most largely used in the production of humans probiotics. For being inhabitants of the natural microbiota, *Lactobacilli* are considered safe microorganisms for the host's health, having low pathogenic potential and lacking the ability to transmit antibiotic resistance factors to pathogenic bacteria [2]. Of note, *Lactobacilli* strains isolated from natural products have been proposed as good probiotic candidates with industrial application. Among these, the strains *Lactobacillus plantarum* 286, isolated from the cocoa fermentation (*Theobroma cacao* L.), and *Lactobacillus plantarum* 81, isolated from the cupuaçu fermentation (*Theobroma grandiflorum*), have been shown to be resistant to simulated gastrointestinal digestion and to be antagonistic to the growth of pathogenic bacteria *in vitro*, including *Listeria monocytogenes* and *Salmonelli* bacteria [3,4].

Studies have shown that the proper administration of certain living microorganisms can provide health benefit to patients suffering from psychiatric illnesses [5]. Microorganisms that fit within this class have been defined as psychobiotics, corresponding to a class of probiotics, mainly of the genus *Lactobacillus* and *Bifidobacterium*, capable of producing neuroactive substances, such as γ-aminobutyric acid (GABA) and serotonin [5]. Psychobiotics have been shown to exert therapeutic effects in clinical studies, having a beneficial effect on mood, anxiety and cognition [6–8]. In pre-clinical studies, administration of probiotics from the genus *Lactobacillus* also decreased anxiety- and depressive-like behaviors in mice [9–12].

The aim of the present study was to investigate the effects of the strains *Lactobacillus plantarum* 286 and *Lactobacillus plantarum* 81 on cognitive, anxiety- and depressive-like behaviors in male mice. Specifically, mice treated chronically with these strains were submitted to locomotor activity evaluation, the forced swim test and the plus-maze discriminative avoidance task.

## Material and methods

### Reactivation and maintenance of strains

The strains of Lactobacillus used in this study were collected from the collection of microorganisms from the Mars Cocoa Center Company (Mars Cocoa, Ilheus, Brazil). The strains *L*. *plantarum* 286 and *L*. *plantarum* 81 were isolated from the natural fermentation of cocoa (*Theobroma cacao*) [3] and cupuaçu (*Theobroma grandiflorum*), respectively [4]. Both were stored under -80°C in glycerol solution, reactivated in commercial MRS broth and cultured at 37°C for 48 hours. Cells were then centrifuged at 10000G/10min, the supernatant was discarded, (0.85%) and solutions were adjusted to 10e10 CFU/mL with sterile saline (0.85%) plus cryoprotectant (15% skim milk). The solutions were distributed in 2 mL cryotubes and stored under freezing at -4°C.

### Subjects

Two-and-a-half-month-old Swiss male mice from our own colony were used. A total of 96 animals were used in this study, being 48 animals per Experiment. Animals were random bred and maintained at the Vivarium of the Universidade Estadual de Santa Cruz (UESC). The animals weighed 25-30g and were separated into groups (8 per cage) in polypropylene cages (32 x 42 x 18 cm) under controlled temperature (22–23°C) and lighting (12:12h light:dark cycle, lights on at 6h45) conditions. Rodent chow (Nuvilab, Quimtia SA, Colombo, PR, Brazil) and water were available *ad libitum* throughout the experiments. At the end of the experiments, animals were euthanized by using a combined i.p. administration of a barbiturate (thiopental at 90 mg/kg) and lidocaine (10 mg/kg) at a volume of 10mg/ml. Animals were maintained according to the National Institutes of Health Guide for the Care and Use of Laboratory Animals (8th Edition, revised 2011) and in accordance with the Brazilian Law for Procedures for Animal Scientific Use (#11794/2008). The Institutional Animal Care and Use Committee of UESC approved the experimental procedures (protocol #012/17).

### Treatment

During the treatment phase, groups were treated daily for 30 days with single doses of 0.1 ml vehicle solution (0.85% saline plus 15% skim milk) or 0.1 ml vehicle solution supplemented with Lp 286 (10^9^/0.1 ml colony-forming units–CFU) or Lp 81 (10^9^/0.1 ml CFU).

### Protocols

#### Open field test (OFT)

Locomotor activity was measured in the open-field, as described by [13]. Briefly, on OFT days, animals were placed in a cylindrical arena with high lateral walls (48 X 50 cm—diameter x height) and the locomotor activity of the animals was recorded with a video camera for 10 min. The arena was cleaned with 5% alcohol/water solution between animals. The total distance traveled, and distance traveled in the center of the arena were quantified using the ANYMAZE® software.

#### Forced swim test (FST)

Mice were subjected to the FST for 6 min in a transparent 5 L beaker containing 4 L of water (23–25⁰C). The test was recorded by a video camera and the latency for immobility was quantified during the test, as well as the immobility time during the final 4 min of the FST using the ANYMAZE® software [14]. After the test, mice were dried with absorbent paper and returned to their home-cages.

#### Plus maze-discriminative avoidance test (PM-DAT)

The PM-DAT was performed as described by Silva and Frussa-Filho [15] and was used to evaluate anxiety-like behavior, memory and learning. By measuring the animals’ activities in the elevated plus maze, this test provides a measure of anxiety-like behavior through the time spent in the open arms of the apparatus. Also, one of the two closed arms is aversive (light and noise stimuli), and during a training session animals learn to avoid that closed arm, learning that can then be measured during a test on the following day in which the aversive stimuli are not presented.

The apparatus was 50 cm from the floor and consisted of 2 open arms and 2 closed arms (total arm length 28.5 cm, arm width 7 cm, arm height (closed arms): 18.5 cm). One of the closed arms provided aversive stimuli when the animal entered it, consisting of light (12,5 W led lamp ≅ 100 lumens) and sound (bell 80 dB). The PM-DAT was divided into training and test sessions. On the training day, animals were placed individually in the center of the apparatus (7 x 7 cm^2^) with free access to all the arms for 10 min, and the aversive stimuli were activated at each entrance of the animal in the aversive closed arm. Twenty-four hours after the training session, the mice were re-exposed to the apparatus for the test session, during which animals had access to all arms of the apparatus for 3 min. The test session was essentially the same as the training session, except that aversive stimuli were not activated. The apparatus was cleaned with 5% alcohol/water solution between animals. The activity of the animals on both the training and test sessions was recorded with a video camera, and the ANYMAZE® software was used to quantify the time spent in each arm of the device.

### Experimental design

The protocols below were established with the goal of investigating whether treatment with the probiotic strains had behavioral effects after the cessation of treatment in order to control for any effects of the treatments on behavior *per se*. For that reason, we conducted our main behavioral tasks (FST and PM-DAT) after treatment cessation, and performed OFT evaluations during the treatment period in order to investigate the effects of ongoing treatment on locomotor activity.

#### Experiment 1: Effects of treatment with Lp 286 and Lp 81 strains on locomotor activity and depressive-like behavior in male mice

**Fig 1:**
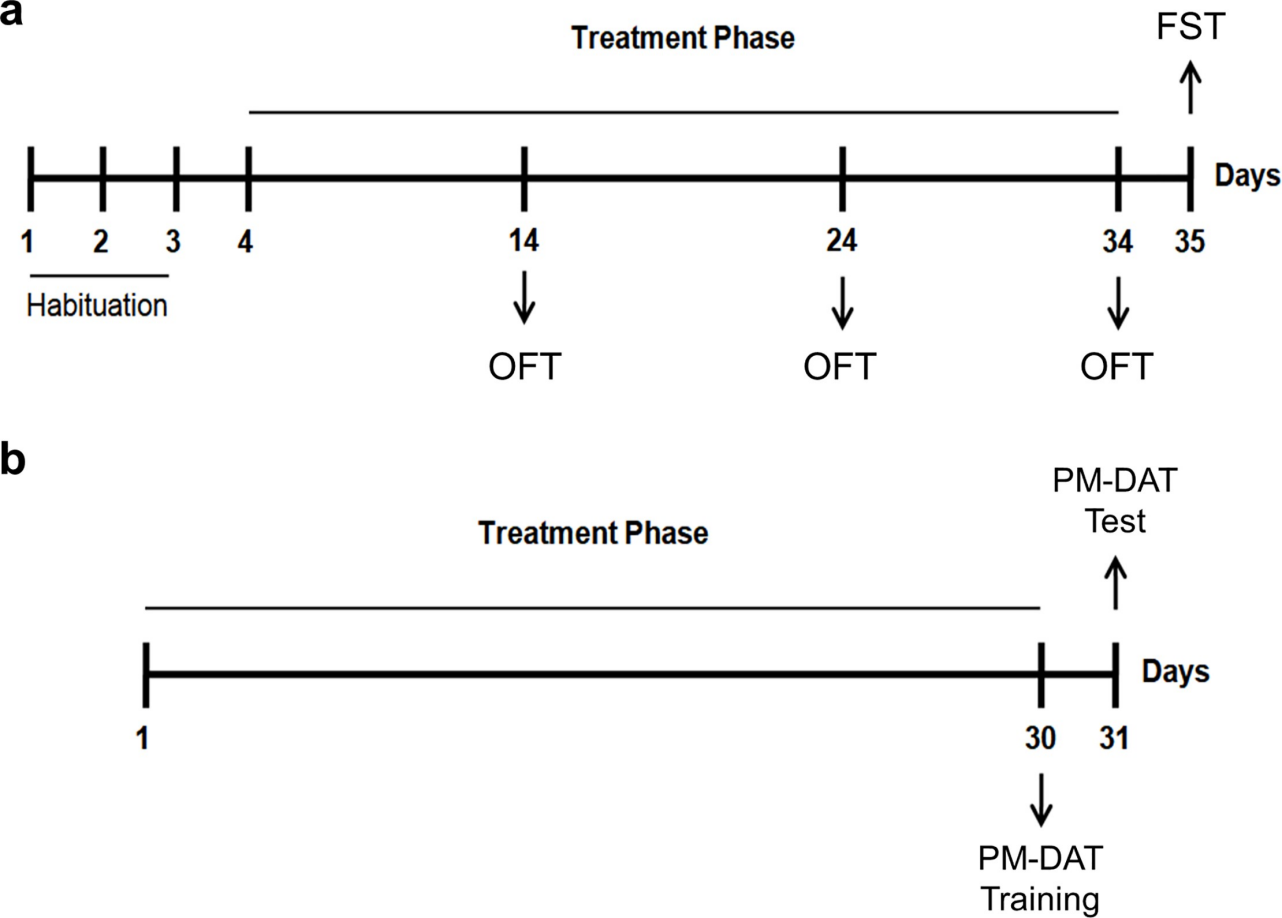
Protocol design across experimental days. On Experiment 1 (a), animals were submitted to 3 days of habituation to the open-field apparatus, and treated (oral gavage) with vehicle, *Lactobacillus plantarum* 286 (Lp 286, 10e9UFC/0.1 mL) or *Lactobacillus plantarum* 81 (Lp 81, 10e9 UFC/0.1 mL) solutions once/day for 30 days. Animals were submitted to the open-field test (OFT) on days 14, 24 and 34, and to the forced swim test (FST) on day 35. On Experiment 2, animals received once/daily administrations (oral gavage) of vehicle, Lp 286 or Lp 81 solutions and were submitted to the training and test sessions of the plus-maze discriminative avoidance test (PM-DAT) on days 30 and 31, respectively.

Fig 1A illustrates the experimental design of Experiment 1. Before the beginning of treatments, animals were submitted to a habituation to the OFT apparatus, being placed in the center of the apparatus for 3 consecutive days. Locomotor activity was quantified on the 3^rd^ day. Following the habituation phase, forty-eight mice were divided into 3 groups (vehicle, Lp 286 and Lp 81, n = 16 per group) and treated during 30 days with once/daily oral (gavage) administration of vehicle, Lp 286 or Lp 81 solutions. During the treatment regimen, animals were submitted to OFT evaluations on days 14, 24 and 34. Twenty-four hours after the last treatment day (Day 35), all animals were submitted to the FST.

#### Experiment 2: Effects of treatment with Lp 286 and Lp 81 strains on anxiety-like behavior, learning and memory in male mice

Fig 1B illustrates the experimental design of Experiment 2. Forty-eight mice were divided into 3 groups (vehicle, Lp 286 and Lp 81, n = 16 per group) and treated during 30 days with once/daily oral (gavage) administration of vehicle, Lp 286 or Lp 81 solutions. On the last treatment day (Day 30), all animals were submitted to the PM-DAT training session, being tested twenty-four hours later (Day 31).

### Statistical analysis

All variables were checked for normality (Shapiro-Wilk test) and homogeneity (Levene test), which validated the use of parametric tests. Multiple comparisons were performed using one- or two-way analysis of variance (ANOVA), with repeated measures (RM) or not. When necessary, Bonferroni's post-hoc test was then performed for multiple comparisons between groups. In all comparisons, a p value below 0.05 was considered as a reference to indicate significant differences.

## Results

The raw data for this study can be found under the DOI: 10.6084/m9.figshare.12168078

### Experiment 1: Effects of treatment with Lp 286 and Lp 81 strains on locomotor activity and depressive-like behavior in male mice

#### Open field test (OFT)

**Fig 2:**
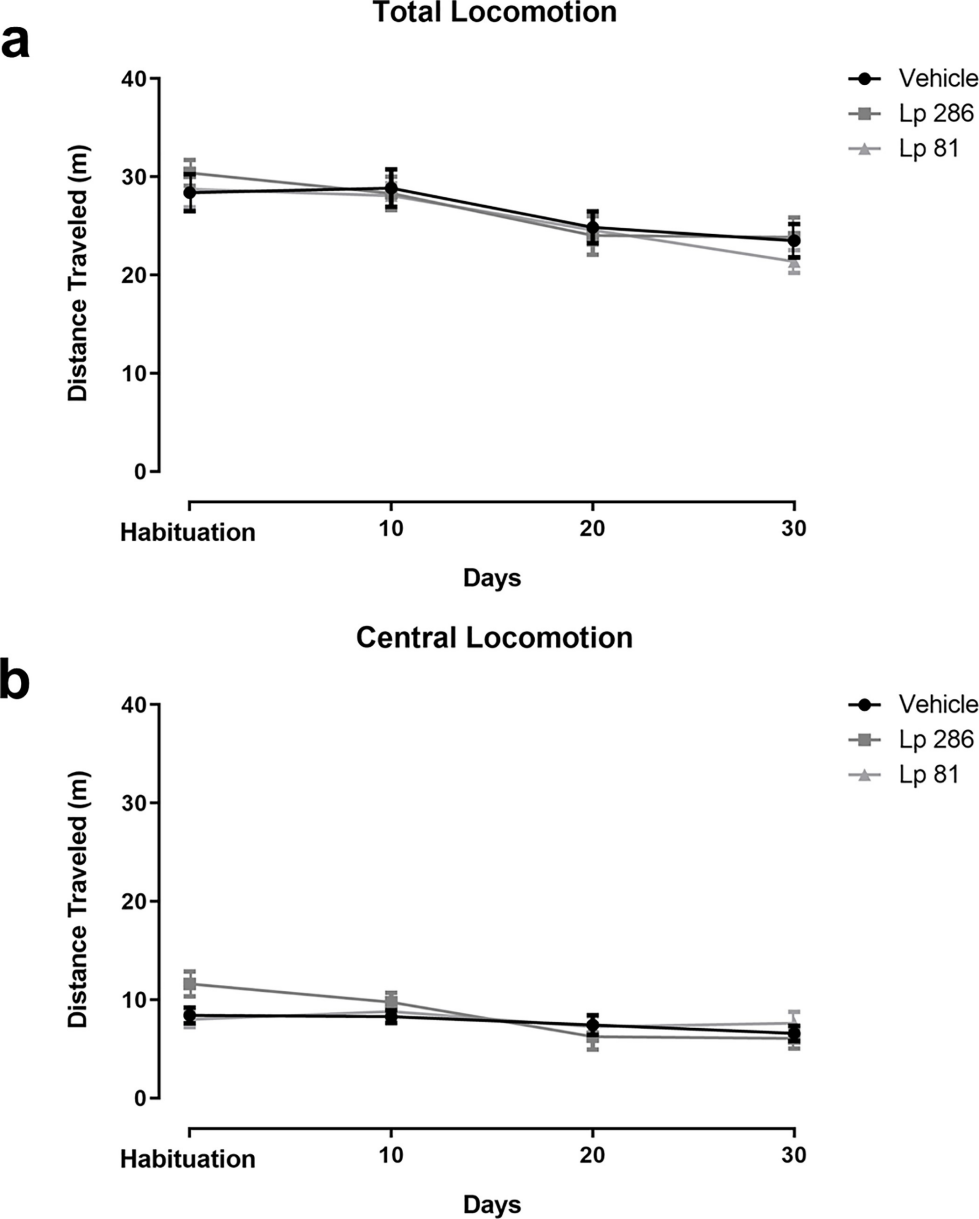
Open-field locomotor activity (distance travelled, m) during habituation and on the 10^th^, 20^th^ or 30^th^ days of treatment (oral gavage) with vehicle, *Lactobacillus plantarum* 286 (Lp 286, 10e9UFC/0.1 mL) or *Lactobacillus plantarum* 81 (Lp 81, 10e9 UFC/0.1 mL) solutions. (a) Total distance traveled and (b) distance traveled in the center of the open-field apparatus. Two-way repeated measures ANOVA showed main effect of time. Data are presented as mean ± SEM.

Analysis of the 3^rd^ habituation session revealed no significant differences between groups (mean±SEM = *Vehicle*: 28.39±1.88; *Lp 286*: 30.39±1.3; *Lp 81*: 28.76±1.86). Two-way RM ANOVA revealed a significant effect of time (Habituation vs Day 10 vs Day 20 vs Day 30) [F(3,45) = 10.90, p<0.0001], but not treatment (vehicle, Lp 286 or Lp 81) [F(2,30) = 0.3169, p = 0.7308] for total distance traveled in the OFT. For the central distance traveled, two-way RM ANOVA revealed a significant effect of time [F(2,135) = 6.100, p<0.001], but not treatment [F(2,45) = 0.4202, p>0.05]. These results suggest that the animals habituated to the OFT apparatus over time, with a decrease in general locomotion due to lack of novelty, and that treatment with the probiotic strains Lp 286 or Lp 81 did not alter general locomotor activity (Fig 2A) or central locomotor activity (Fig 2B).

#### Forced swim test (FST)

**Fig 3:**
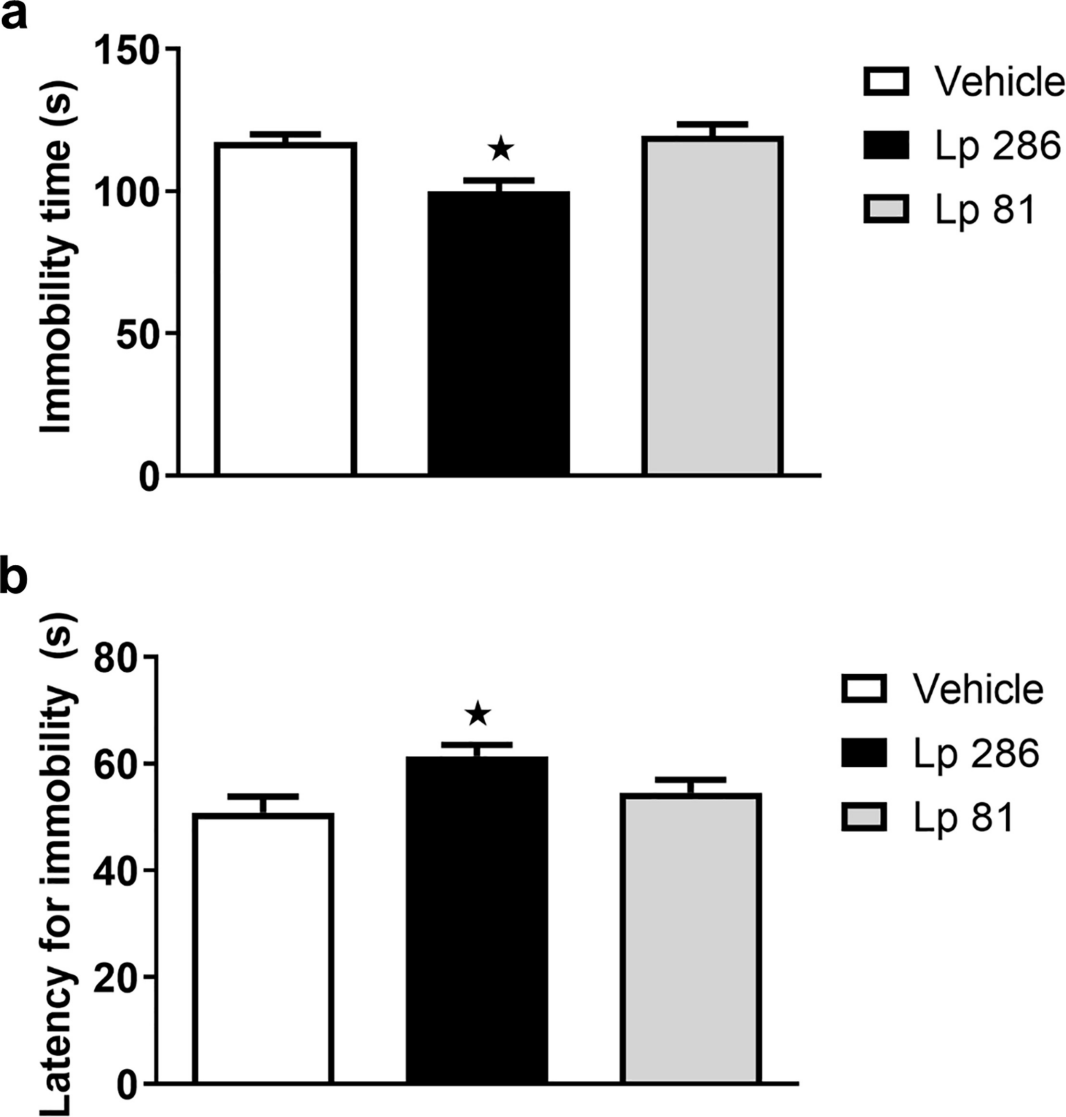
(a) Immobility time (b) and latency for immobility during the forced swim test performed after 30 days of once/daily treatment (oral gavage) with vehicle, *Lactobacillus plantarum* 286 (Lp 286, 10e9UFC/0.1 mL) or *Lactobacillus plantarum* 81 (Lp 81, 10e9 UFC/0.1 mL) solutions. Data are presented as mean ± SEM. One-way ANOVA followed by Bonferroni t-test. *p<0.05 compared to the vehicle group.

In the FST, significant differences were observed between groups for the immobility time (one-way ANOVA, [F(2,45) = 8.941, p<0.001]) and latency for immobility (one-way ANOVA, [F(2,45) = 4.474, p<0.05]). Animals treated with the strain Lp 286, but not the strain Lp 81, presented a significant reduction in the immobility time (Fig 3A) and an increase in the latency for immobility (Fig 3B) during the FST compared to vehicle-treated animals (Bonferroni *post hoc* test, p <0.05).

### Experiment 2: Effects of treatment with Lp 286 and Lp 81 strains on anxiety-like behavior, learning and memory in male mice

#### Plus maze—discriminative avoidance test (PM-DAT)–training session

**Fig 4:**
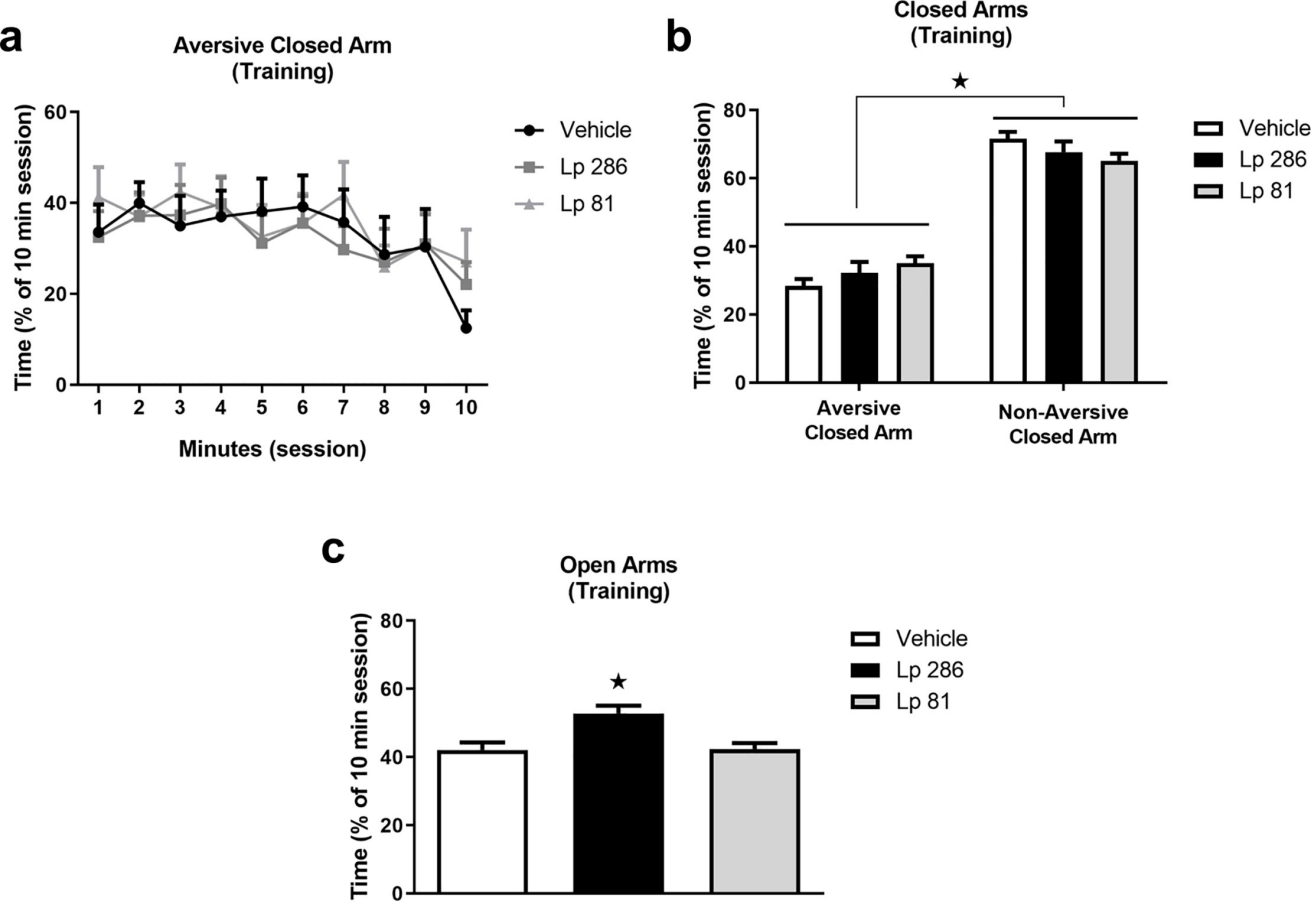
Time spent in the different arms of the modified elevated plus-maze during the training session of the plus-maze discriminative avoidance test. Training was performed on the 30^th^ day of once/daily treatment (oral gavage) with vehicle, *Lactobacillus plantarum* 286 (Lp 286, 10e9UFC/0.1 mL) or *Lactobacillus plantarum* 81 (Lp 81, 10e9 UFC/0.1 mL) solutions. (a) Time spent in the aversive closed arm over the 10-minute training session. (b) Comparison of the time spent in the aversive vs non-aversive closed arms during the 10-minute training session. (c) Time spent in the open arms during the 10-minute training session. Data are expressed as % of the 10-minute session spent in each arm of the apparatus, and presented as mean ± SEM. One- or two-way ANOVA followed by Bonferroni t-test. *p<0.05 compared to the (b) aversive closed arm or to the (c) vehicle group. Two-way repeated measures ANOVA showed a significant effects of time for (a).

When analyzing the time spent in the aversive closed arm over the 10 min training session, a significant effect of time (minutes 1 through 10), but not treatment, was observed [F(9,405) = 2.640, p<0.01], indicating that all groups equally learned to avoid the aversive closed arm within the training session (Fig 4A). When analyzing the difference between the time spent in the two closed arms during the training day, a significant effect of compartment (aversive vs non-aversive closed arms), but not treatment (vehicle, Lp 286 or Lp 81) was observed (two-way RM ANOVA, F(1,45) = 162.6, p<0.0001), indicating that animals spent more time in the non-aversive closed arms compared to the aversive closed arms, regardless of previous treatment (Fig 4B).

When analyzing the time spent in the open arms during the training session, a significant effect of treatment was observed (one-way ANOVA, [F(2,45) = 8.395, p<0.001]. The group treated with the Lp 286 strain, but not with the Lp 81 strain, spent a significantly longer time in the open arms compared to the vehicle group, indicative of an anxiolytic-like effect (Fig 4C, Bonferroni t-test, p<0.05).

No significant differences were observed between groups for the number of entries into the aversive closed arm (S1 Fig), the number of entries into the non-aversive closed arm (S2 Fig) or the number of entries into the open arms (S3 Fig) during the training session.

#### PM-DAT–test session

**Fig 5:**
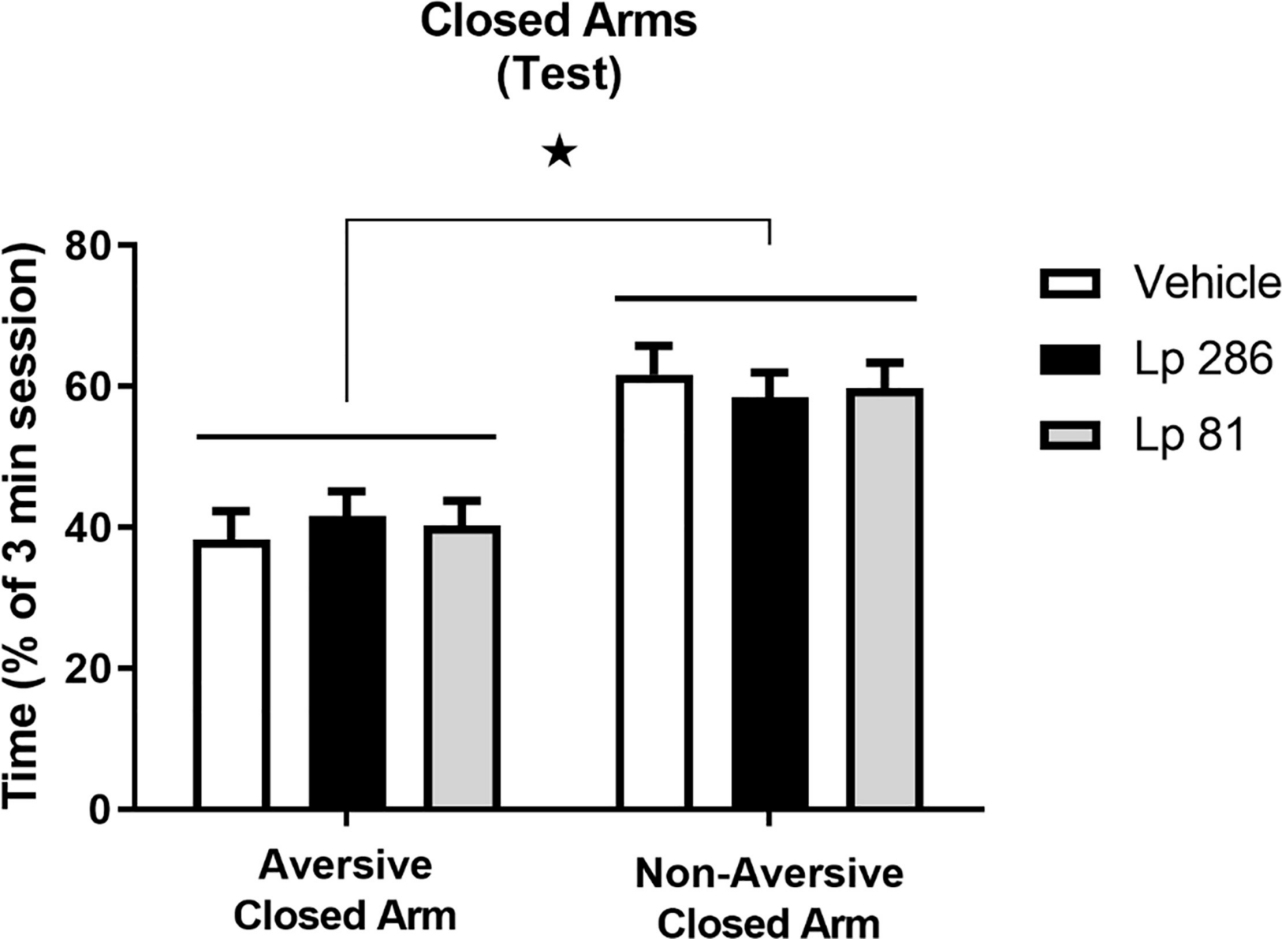
Time spent in the aversive vs non-aversive closed arms of the modified elevated plus-maze during the test session of the plus-maze discriminative avoidance test. Test was performed on the day after the training session after once/daily treatment (oral gavage) with vehicle, *Lactobacillus plantarum* 286 (Lp 286, 10e9UFC/0.1 mL) or *Lactobacillus plantarum* 81 (Lp 81, 10e9 UFC/0.1 mL) solutions. Comparison of the time spent in the aversive vs non-aversive closed arms during the 3-minute test session. Data are expressed as % of the 3-minute session spent in each arm of the apparatus, and presented as mean ± SEM. Two-way repeated measures ANOVA followed by Bonferroni t-test. *p<0.05 compared to the aversive closed arm.

When analyzing the difference between the time spent in the two closed arms during the test day, a significant effect of compartment (aversive vs non-aversive closed arms), but not treatment (vehicle, Lp 286 or Lp 81) was observed (two-way RM ANOVA, [F(1,15) = 13.29, p<0.01]), indicating that animals spent more time in the non-aversive closed arms compared to the aversive closed arms, regardless of previous treatment (Fig 5).

No significant differences were observed between groups for the number of entries into the aversive closed arm (S4 Fig) or the number of entries into the non-aversive closed arm (S5 Fig) during the test session.

## Discussion

Probiotic bacteria have been proposed to influence systems beyond the gastrointestinal (GI) tract and confer several benefits to the host [16–18]. In the present study, we demonstrate for the first time that the probiotic strain *Lactobacillus plantarum* 286, but not the strain *Lactobacillus plantarum* 81, exerted anti-depressant- and anxiolytic-like effects in male mice without altering general locomotor activity or memory consolidation/retrieval. These findings add to the body of evidence suggesting that probiotics can serve as adjuvants for the treatment of psychiatric disorders, including studies showing that the administration of other probiotic strains from the genus *Lactobacillus* decreased anxiety- and depressive-like behaviors in mice [9–12]. Our study is the first to show that *Lactobacillus plantarum* 286, a probiotic strain isolated from the cocoa (*Theobroma cacao* L.) fermentation, may have therapeutic effects beyond those related to the GI tract [3,4].

Our results on the anti-depressant-like effects of the strain *Lactobacillus plantarum* 286 corroborate studies showing similar effects after the administration of the *Lactobacilli* strains *L*. *rhamnosus* JB-1 [9], *L*. *helveticus* NS8 [10] and *L*. *acidophilus* LAB/LAB FB [19]. These findings are also in agreement with a clinical study showing that probiotic consumption (*Lactobacillus casei* Shirota) positively regulated mood in healthy and depressed individuals [7]. Although the mechanisms underlying these effects remain unknown, several properties of probiotic bacteria could contribute to their anti-depressant effects. For instance, kynurenine is converted in the brain into its metabolites (kynurenic acid and quinolinic acid), which cause neuroinflammation [20,21] and have been associated with depression [20, 22,23]. Bacteria from the genus *Lactobacillus* are known to produce reactive oxygen species that are capable of suppressing the metabolism of kynurenine in the host [24]. Therefore, it could be hypothesized that inhibition of kynurenine metabolism could contribute to probiotic-induced anti-depressant effects. However, whether Lp286 specifically can block kynurenine metabolism remains to be investigated.

Pre-clinical data have also shown that commensal bacteria synthesize GABA [25,26], serotonin, dopamine, noradrenaline and acetylcholine [26] as secondary products of their metabolism, which could also be theorized to contribute to their positive effects on depression and anxiety models observed in the present study. In addition, administration of the probiotic *Lactobacillus* GG has been shown to increase plasma IL-10 concentration [27]. Conventional antidepressants are also known to increase IL-10 levels, with IL-10 being a potent immunoregulatory interleukin capable of suppressing inflammation and changes in the central nervous system (CNS) associated with depression [28]. Although no mechanisms potentially responsible for the behavioral effects of Lp286 have been investigated in the present study, previous studies on the mechanisms of action of other *Lactobacilli* bacteria further emphasize that probiotic bacteria could be exerting behavioral effects through many different mechanisms.

Our studies using the plus maze-discriminative avoidance test [15] demonstrated that the strains Lp 286 and Lp 81 did not alter learning and memory. Similarly, Nimgampalle and Kuna [29] also demonstrated that the administration of *Lactobacillus plantarum* did not negatively alter cognition in rodents using other models. However, our studies did demonstrate an anxiolytic-like effect of the strains Lp 286, shown by the fact that animals treated with this strain spent more time in the open arms of the elevated plus maze apparatus compared to vehicle-treated animals, a classic effect interpreted as a decrease in anxiety-like behavior [30,31]. It is important to note that during the treatment protocol, ongoing treatment with Lp 286 did not alter central locomotion in the open-field, which is another commonly used measure of anxiety-like behavior. These findings suggest that there seems to be a selective effect of treatment cessation on behavior, either due to the physiological effects of the bacteria or due to cessation of oral gavage.

The findings indicating that treatment with Lp 286 induced anxiolytic-like effects in the PM-DAT are in agreement with the study by Bravo et al. [9] showing that the chronic administration of *Lactobacillus rhamnosus* JB-1 resulted in a greater number of entries in the open arms of the elevated plus-maze in mice. In their study, Bravo and colleagues [9] demonstrate that GABAergic mechanisms are involved in the anxiolytic-like effects of *L*. *rhamnosus* JB-1. Bacteria of the genus *Lactobacillus* have, indeed, been shown to break down glutamate, leading to an increase in GABA levels in the GI tract and, consequently, in the CNS [25], which could also account for their anxiety-reducing effects. Therefore, findings from previous studies suggest that the anxiety-decreasing effects of *Lactobacilli* bacteria may be mediated by GABA neurotransmission, and future studies should focus on the investigation of the role of GABA on Lp286-induced anxiolytic effects.

In summary, our findings suggest that the new strain of *Lactobacilli* bacteria *Lactobacillus plantarum* 286 extracted from the fermentation of cocoa may have anxiolytic and anti-depressant effects. These findings corroborate many others showing that probiotic bacteria exert beneficial effects in anxiety and depression, including clinical studies [6]. It is important to point out, however, that in our study another strain of *Lactobacilli* bacteria, *Lactobacillus plantarum* 81, did not exert effects on mouse models of anxiety- and depressive-like behaviors. Also noteworthy is the fact that the specific strain of Swiss mice used in the present study shows lower anxiety-like behavior compared to other strains of Swiss mice [32], which could have contributed to the lack of effect of Lp81 in the present study. Therefore, more robust effects may have been obtained had we used a high-anxiety strain of mice. Understanding how these and other strains of bacteria with distinct behavioral effects differ in terms of their physiological effects in varying strains of animals may further elucidate the mechanisms through which probiotic bacteria can decrease anxiety and depression.

## Supporting information

S1 Fig Number of entries into the aversive closed arm of the apparatus during the *training* session of the plus maze—discriminative avoidance test. ANOVA: [F(2,45) = 0.4426; p = 0.6451].
(PDF)

S2 Fig Number of entries into the non-aversive closed arm of the apparatus during the *training* session of the plus maze—discriminative avoidance test. ANOVA: [F(2,45) = 1.543; p = 0.2249].
(PDF)

S3 Fig Number of entries into the open arms of the apparatus during the *training* session of the plus maze—discriminative avoidance test. ANOVA: [F(2,45) = 0.2252; p = 0.7992].
(PDF)

S4 Fig Number of entries into the aversive closed arm of the apparatus during the *test* session of the plus maze—discriminative avoidance test. ANOVA: [F(2,45) = 0.3527; p = 0.7047].
(PDF)

S5 Fig Number of entries into the non-aversive closed arm of the apparatus during the *test* session of the plus maze—discriminative avoidance test. ANOVA: [F(2,45) = 1.924; p = 0.1578].
(PDF)
