# Supplementary material for: Effects of chronic treatment with new strains of Lactobacillus plantarum on cognitive, anxiety- and depressive-like behaviors in male mice
Source: PLoS One. 2020 Jun 19;15(6):e0234037. doi: 10.1371/journal.pone.0234037 (PMC7304620; doi:10.1371/journal.pone.0234037)
Supplement: S2 Fig — ANOVA: [F(2,45) = 1.543; p = 0.2249]. (PDF) [file pone.0234037.s002.pdf]

Supplementary material for the article *"Effects of chronic treatment with new strains of Lactobacillus plantarum on cognitive, anxiety- and depressive-like behaviors in male mice"*

Experiment 2: Effects of treatment with Lp 286 and Lp 81 strains on anxiety-like behavior, learning and memory in male mice

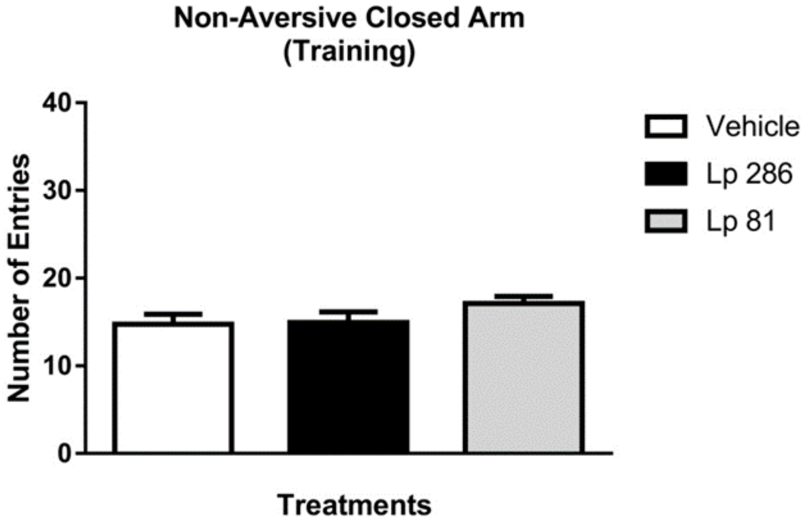

S2 Figure. Number of entries into the non-aversive closed arm of the apparatus during the *training* session of the plus maze - discriminative avoidance test. ANOVA: [F(2,45)=1.543; p=0.2249].
